# Exercise and manual auricular acupuncture: a pilot assessor-blind randomised controlled trial. (The acupuncture and personalised exercise programme (APEP) Trial)

**DOI:** 10.1186/1471-2474-9-31

**Published:** 2008-03-06

**Authors:** SM McDonough, SD Liddle, R Hunter, DM Walsh, P Glasgow, G Gormley, D Hurley, A Delitto, J Park, I Bradbury, GD Baxter

**Affiliations:** 1 Health and Rehabilitation Sciences Research Institute and School of Health Sciences, University of Ulster, Jordanstown, Northern Ireland, UK; 2 Sports Medicine, Sports Institute Northern Ireland, Jordanstown, Northern Ireland, UK; 3 Department of General Practice, Queen's University Belfast, Belfast, Northern Ireland, UK; 4 School of Physiotherapy and Performance Sciences, University College Dublin, Dublin, Ireland; 5 Department of Physical Therapy, University of Pittsburgh, Pittsburgh, USA; 6 Department of Physical Medicine and Rehabilitation, University of North Carolina, Chapel Hill, USA; 7 Frontier Science Ltd, Inverness-shire, UK; 8 Centre for Physiotherapy Research, University of Otago, Otago, New Zealand

## Abstract

**Background:**

Evidence supports the use of exercise for chronic low back pain (CLBP); however, adherence is often poor due to ongoing pain. Auricular acupuncture is a form of pain relief involving the stimulation of points on the outer ear corresponding with specific body parts. It may be a useful adjunct to exercise in managing CLBP; however, there is only limited evidence to support its use with this patient group.

**Methods/Design:**

This study was designed to test the feasibility of an assessor-blind randomised controlled trial which assess the effects on clinical outcomes and exercise adherence of adding manual auricular acupuncture to a personalised and supervised exercise programme (PEP) for CLBP. No sample size calculation has been carried out as this study aims to identify CLBP referral rates within the catchment area of the study site. The researchers aim to recruit four cohorts of n = 20 participants to facilitate a power analysis for a future randomised controlled trial. A computer generated random allocation sequence will be prepared centrally and used to allocate participants by cohort to one of the following interventions: 1) six weeks of PEP *plus *manual auricular acupuncture; 2) six weeks of PEP alone. Both groups will also complete a further six weeks of self-paced exercise with telephone follow-up support. In addition to a baseline and exit questionnaire at the beginning and end of the study, the following outcomes will be collected at baseline, and after 7, 13 and 25 weeks: pain frequency and bothersomeness, back-specific function, objective assessment and recall of physical activity, use of analgesia, perceived self-efficacy, fear avoidance beliefs, and beliefs about the consequences of back pain. Since this is a feasibility study, significance tests will not be presented, and treatment effects will be represented by point estimates and confidence intervals. For each outcome variable, analysis of covariance will be performed on the data, conditioning on the baseline value.

**Discussion:**

The results of this study investigating the adjuvant effects of auricular acupuncture to exercise in managing CLBP will be used to inform the design of a future multi-centre randomised controlled trial.

**Trial Registration:**

Current Controlled Trials ISRCTN94142364.

## Background

Current research evidence supports the use of exercise-based treatment programmes for CLBP that encourage the patient to assume an active role in their recovery [1-3]. Outcomes are also claimed to improve when exercise is supervised and personalised/individually tailored [1,3-5]. One of the main factors limiting the success of such an active approach is the patient's level of adherence, which often suffers as a result of exercise-induced exacerbations in pain [2,3,5,6]. People with CLBP typically lack faith in the recommendation to stay active *despite *pain [7], with the result being an avoidance of activity in order to avoid pain. The results of a recent prospective cohort study [8] have indicated that people with CLBP who experienced a reduction in pain during treatment also experienced the greatest functional improvements. The benefits of adequate pain control may be expected to contribute to the effectiveness of exercise-based intervention, by increasing levels of activity and self-confidence, and modifying pain perception and disability [5]; thus there is the potential for an adjunctive pain relieving modality for exercise therapy.

Of the various complementary and alternative medicine (CAM) available for pain relief, acupuncture has become increasingly accepted as an effective means of pain control, due to its holistic approach and limited side effects [9,10]. Current evidence supports the use of acupuncture for people with CLBP, particularly when provided alongside other conventional therapies [10-13]. Thomas and colleagues [13] concluded that acupuncture was significantly more effective in reducing bodily pain and participants' concerns about back pain than usual care for up to 24 months. A recent study has suggested that stimulation of auricular (ear) acupuncture points is effective for the treatment of CLBP [14]: this study compared electrical stimulation of auricular points with manual stimulation, and found that both groups experienced pain relief. Aside from the above study and the use of auricular acupuncture for pain relief for cervical spine pain [15,16] and after knee and hip surgery [17,18], the evidence for auricular acupuncture is limited and there have been no studies that have examined the adjuvant effect of auricular acupuncture to an exercise programme in CLBP. Auricular acupuncture is relatively easy to administer, promoting pain relief with minimal interruption to the individual's normal daily activities. Needles can stay *in situ *for up to seven days allowing participants to self-treat at home. It is proposed that the addition of AA to a supervised PEP will address the pain relieving expectations of participants, and help to decrease the barriers to exercise that can often limit adherence. The individually tailored approach to exercise is intended to foster the development of active self-management strategies, and functionally-related goals that are the necessary pre-requisites for effective long-term symptom management.

The aim of this study is to evaluate the feasibility of a randomised controlled trial (RCT) exploring the effects on clinical outcomes and exercise adherence of adding manual AA to a PEP, when compared to the PEP alone, for participants with CLBP using the MRC framework for the design and evaluation of complex interventions [19].

## Methods/Design

### Study design

An assessor-blind randomised controlled trial with six-month follow-up. Ethical approval was obtained from the Northern Ireland Office for Research Ethics Committee.

### Participants

Individuals diagnosed with CLBP who fulfil the inclusion/exclusion criteria (see Table 1) will be recruited between April 2007 and September 2008 to attend classes in a purpose built gym at the University of Ulster, Jordanstown, Northern Ireland. Several approaches to recruitment will be undertaken, via healthcare professionals and via the general public. Prospective and retrospective recruitment from General Practitioner (GP) practices (n = 14) within the catchment area of the University of Ulster, plus identification of people with CLBP from the physiotherapy waiting lists (referral via GP) in a large Healthcare Trust, close to the University, will be used. GPs willing to participate will receive a full explanation (verbal and written) of the trial procedure, following which their agreement to participate will be confirmed in writing. The physiotherapy managers of local primary care trusts, typically receiving GP referrals for CLBP, will also receive a full explanation (verbal and written) of the intended trial dates and procedures. Finally, the feasibility of recruitment from the general public, and appropriateness of referrals via this route, will be tested via poster/email advertisements and a website [20] to staff and students at the University of Ulster. For this method of recruitment, the relevant GP's contact details, supplied by staff and students, will be used to confirm the medical suitability of each participant for our trial.

**Table 1:** Inclusion/exclusion criteria

**Inclusion criteria**	**Exclusion criteria**
Participants with chronic (≥3 months) or recurrent (≥3 episodes in previous 12 months) LBP of mechanical origin with/without radiation to the lower limb	Currently or having received treatment for CLBP within the previous 3 months
Males/females between 18–65 years	Red flags indicating serious spinal pathology, e.g. cancer, cauda equina lesion
No spinal surgery within the previous 12 months	Radicular pain indicative of nerve root compression **
Participants deemed suitable by their GP to carry out an exercise programme	Participants diagnosed with severe spinal stenosis, spondylolisthesis, fibromyalgia
Participants deemed suitable by their GP to receive acupuncture treatment	History of systemic/inflammatory disease, e.g. rheumatoid arthritis
Participants willing to attend for a 6-week treatment programme of exercise and manual AA	Concomitant medical condition that contraindicates acupuncture
Fluency in English (verbal and written)	Participants with acute (< 6 weeks) or subacute LBP (6–12 weeks), provided that they have experienced < 3 LBP episodes during the previous 12 months
Access to a telephone (for follow-up support)	Previously received auricular acupuncture
Participants categorised as 'low' or 'moderate' activity levels on the International Physical Activity Questionnaire (IPAQ)	Participants with any confounding conditions such as a neurological disorder or currently receiving treatment for cancer
	Road traffic accident causing LBP
	History of psychological or psychiatric illness
	Participants having multiple body and/or ear piercings
	Fear of needles

** In accordance with the Clinical Standards Advisory Group (1994) [21] and the Royal College of General Practitioners Guidelines (Waddell et al., 1999) [22], participants presenting with any or all of the following criteria indicative of radicular pain will be excluded from the study:

(a) unilateral pain usually worse than back pain;

(b) pain generally radiating to the foot or toes;

(c) numbness or paraesthesia in the same distribution;

(d) reduced straight leg raise that produces leg pain;

(e) motor, sensory or reflex change limited to one nerve root.

**Figure 1:**
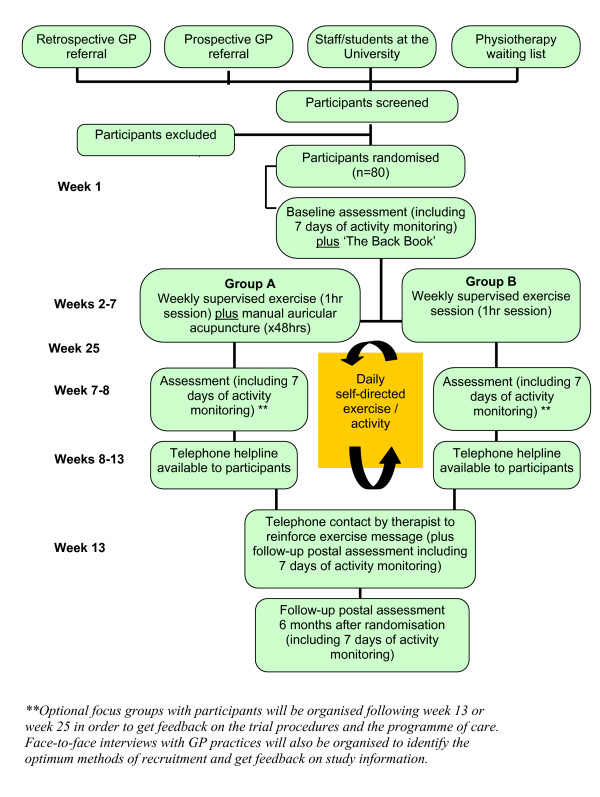
Recruitment of participants and flow through trial.

For all methods of recruitment, once we receive confirmation that they are medically fit to participate in the trial, participants will receive a trial information pack from the lead researcher, be screened by phone, and if eligible an appointment will be made for baseline assessment one week later. During this appointment, the trial procedures will be explained in detail and written informed consent will be obtained. Participants who provide written informed consent will then be randomised, by cohort, according to Figure 1. In order to investigate whether treatment preference has any influence on outcomes, prior to randomisation each participant will be asked which treatment (A or B) he/she would prefer to receive.

### Interventions

#### Personalised and supervised Exercise Programme (PEP)

##### Baseline Assessment and Goal Setting

Prior to the start of the PEP, each participant will attend the University's Health and Rehabilitation Sciences Research Institute (Jordanstown campus) for a baseline assessment (week 1, Figure 1). This will consist of: an individual consultation with one of two chartered physiotherapists (to discuss the content and aims of the PEP); an assessment of the participant's current level of activity, exercise capabilities and perceived barriers to recovery; recording of the participant's treatment expectations, and any previous CAM treatment; a brief lumbar spine assessment to rule out specific LBP (see Table 1 for exclusion criteria for radicular pain) [21,22]; discussion and agreement, between the therapist and the participant, on short and long-term treatment goals. The aim of agreeing such goals is to gradually include activities or postures which the participant has been avoiding because of their LBP, as well increasing their general activity/exercise levels. Each participant will be asked to document their agreed short term and long term goals in their logbook and the exercise class Record Card. The physiotherapist will emphasise to the participants that the supervised PEP classes are a stepping-stone to self directed activity and will emphasise some of key messages from the Back Book (see below) on how to self manage their back pain.

##### Exercise Class Format

The PEP element of the intervention, facilitated by the physiotherapists, will follow a group-based format (maximum participants per group, n = 10) similar to the 'Back to Fitness' programme [23] used in the recently published MRC-funded UK BEAM trial [24]: two of the research team (DB, PG) are experienced in this approach as coordinators of the Northern Ireland Regional Centre for the UK BEAM trial. Participants will attend the Research Institute once a week for 6 weeks for a supervised group exercise session lasting for one hour. The exercise programme will consist of a 10-minute warm-up, a combination of core strengthening, flexibility and cardiovascular exercise using a series of 'exercise stations', and a 10-minute cool down and period of relaxation. The added benefit of this particular programme is that the physiotherapist is available to monitor, advise and encourage participants according to their individual treatment goals and exercise capabilities, and to help participants identify which exercise(s) they could realistically continue independently of the treatment sessions, i.e. foster the development of self-management strategies, and improve self-efficacy. Physiotherapists will also advise participants on correct exercise technique, and review and update treatment goals accordingly as the programme progresses. To identify and combat illness behaviours, during the classes physiotherapists will use a biopsychosocial approach to management, based on cognitive behavioural therapy (CBT) principles [23,24]. Each participant will monitor their own progress by documenting exercise progression in their Exercise Class Record Card during each class. This Record Card will also act as a reminder of their individual short term and long term goals, which can be updated with the physiotherapist during the six week class.

##### Education component

There is no separate education intervention/session in this study. Instead, various educational components will be incorporated into the main intervention using a CBT approach. During the baseline assessment each participant will receive a copy of 'The Back Book' [25], to reinforce the message to remain active despite pain, and develop positive coping strategies in the event of an exacerbation of symptoms. Messages from 'The Back Book' [25] will be placed on the walls in the gym and waiting area in order to reinforce key points. During each exercise session the participants will be given a 'Tip for the Day' based on the 'Back to Fitness' programme.

At the beginning of the six week exercise intervention, each participant will receive a home exercise programme containing photographs of the exercises performed during the class, along with information on warm up and cool down. Exercise/activity will gradually be incorporated into the individual's daily routine, as identified in their short and long-term goals during their baseline assessment. Participants will be encouraged to accept responsibility for determining and carrying out their weekly programme of activity. Adherence with the supervised exercise programme will be recorded as the number of sessions attended. Adherence with activity outside of the supervised exercise session will be captured using an activPAL™ professional physical activity logger (PAL technologies, Glasgow, UK) [26] and a structured activity logbook completed by the participant.

#### Telephone helpline

At the end of the structured exercise ± manual auricular acupuncture treatments (week 7), participants will be advised to continue with daily self-directed exercise/activity as agreed with the therapist. A free telephone helpline will also be available three times per week for a two-hour period for a further six weeks for participants who feel that they need advice or support from the therapist, or to answer any other queries or concerns participants may have. Participants' use of this helpline will be closely monitored and calls recorded and at the end of this phase (week 13), each participant will receive a telephone follow-up call from their therapist to provide advice on exercises and functional activities as appropriate, to reinforce the value of exercise adherence, and if necessary to re-evaluate treatment goals.

#### Auricular acupuncture

**Figure 2:**
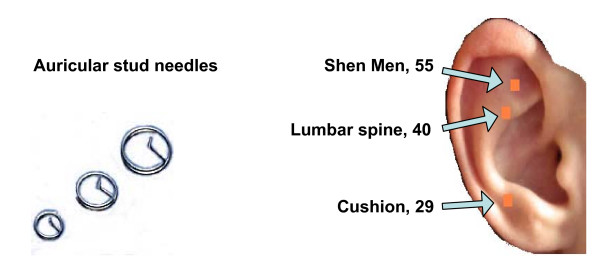
**Auricular acupuncture needles and their placement on the outer ear.** Image of auricular needles courtesy of Scarborough Acupuncture supplies, Somerset, United Kingdom.

Prior to each exercise class, participants in treatment group A will receive manual AA (using conventional auricular stud needles). The stud needles consist of a vertical shaft which inserts into the ear, and an external component which is a horizontal circular piece of metal that sits flat onto the surface of the ear; this flat circle is then covered with a small plaster. For each participant receiving manual AA, a stud needle will be inserted at three specific auricular acupuncture points (see Figure 2). Participants will be asked to manually stimulate the needles every three waking hours, or as required for their pain, and to record this in their daily activity logbook. They will be asked to remove the needles after 48 hours [14] and retain for safe disposal by the therapist during the next treatment session.

#### Experience and Training of Physiotherapists

Two chartered physiotherapists with experience in musculoskeletal physiotherapy and acupuncture will carry out all treatments. Only physiotherapists who have recognised acupuncture training and are members of Association of Acupuncturists in Chartered Physiotherapy (AACP) will be recruited. In addition, each physiotherapist will undertake 3.5 days of training on auricular acupuncture, baseline assessment (including goal setting), and practicalities of running the exercise class. Physiotherapists will be trained in CBT principles by a member of the UK BEAM trial [24] research team (PG). Treating physiotherapists will meet with the research team on a monthly basis to provide an update on the stage of the trial and highlight any potential issues. A member of the research team will monitor the PEP baseline assessments, exercise classes and acupuncture interventions on an ongoing basis.

### Aim and Objectives

To evaluate the feasibility of an RCT exploring the effects on clinical outcomes and exercise adherence of adding manual AA to an individually tailored and supervised exercise programme, when compared to the exercise programme alone.

To determine the feasibility of the trial procedures and establish the most efficient and effective design for a larger RCT by:

(i) Identifying the rate of participation and referrals from the various recruitment routes;

(ii) Determining the actual numbers recruited, adherence and drop out;

(iii) Piloting the methodological procedures;

(iv) Identifying participants' use of a free telephone advice and support service;

(v) Completing a qualitative exploration of the trial procedures and design;

(vi) Confirming training and monitoring requirements for a main trial; and,

(vii) Identifying a crude approximation of the effect size of each treatment package.

### Outcome measures

A number of outcome measures will be collected at week 1 (baseline), and week 7 (end of scheduled exercise ± manual AA) by a blinded assessor as part of the scheduled intervention, and by post at week 13 and at 6 months after initial randomisation (except for the Readiness to Change Questionnaire, the Holistic Complementary and Alternative Health Questionnaire and the Exit Questionnaire). The uptake of the free telephone advice and support service, available between weeks 8–13, will be monitored to establish the value of such a service in a future RCT. Participants in treatment group A will be asked to record in their daily activity logbook how often they manually stimulated the stud needles during the 48 hour treatment period, and all participants will also be asked to record any changes in their analgesic intake during the trial. All outcome measures data will be securely stored and analysed once the trial is complete. Treating physiotherapists will be made aware of the results of the Readiness to Change, Back Beliefs, Fear Avoidance Beliefs Questionnaires and the General Perceived Self-Efficacy Scale, in order to adapt the PEP accordingly.

#### Baseline Questionnaire

This has been developed by the research team to collect information from participants concerning any previous CAM treatment received, any perceived difficulties in adhering to exercise for their low back pain, and their treatment expectations.

#### Readiness to Change Questionnaire

Participants are asked to assess their current levels of physical activity participation. This questionnaire [27] will be collected at week 1 (baseline) only, and used as a predictor of compliance.

#### Visual Analogue Scale (VAS) (0–10 cm)

The VAS is widely used within the clinical setting, and demonstrates acceptable reliability, particularly when used with the same individual [28]. Since such a scale only assesses one dimension of pain, it is recommended that both the bothersomeness and intensity of pain are captured within the assessment, and an average score recorded [28,29]. Participants will be asked to rate their level of back pain and leg pain on average over the past week.

#### Oswestry Disability Questionnaire (ODQ)

The ODQ [30] is a valid and reliable measure of pain and physical function in those with LBP [29-31]. It consists of 10 sections, each with six levels that assess the individual's limitations in various activities of daily living (maximum score in each section = five points): the sum of all sections is divided by the total possible score and the result multiplied by 100 to generate a percentage disability score. Values range from 0 (best health state) to 100 (worst health state) with an average score of 43% identified for chronic back pain participants [30,31]. A minimum clinically important change of 4% has been recommended [32].

#### International Physical Activity Questionnaire (IPAQ-Short Form)

This is a self- or telephone-administered physical activity recall questionnaire, which asks the participant about the time they spent being physically active in the last seven days [33]. Extensive reliability and validity testing across 12 countries indicate that it is a viable method of monitoring population levels of physical activity globally for populations of 18–69 years of age.

#### Physical activity monitoring (ActivPAL™ Professional Physical Activity Logger, PAL technologies, Glasgow, UK)

This is an activity monitoring device that is capable of recording steps taken, cadence, time spent lying/sitting, standing and stepping under free-living conditions [34,35]. The ActivPAL™ professional physical activity logger is a small credit card sized device worn on the anterior thigh. Participants will be shown how to attach the device on the anterior aspect of their dominant thigh, at exactly one third of the distance between the superior pole of the patella and the anterior superior iliac spine, by the outcome assessor. The device will be attached using PALstickies™ and reinforced with Vulcan fixation tape (Mobilis Healthcare Group Limited, Oldham, Lancashire, UK). Exact replacement of the monitor will be assured by using a semi-permanent pen to mark above and below the monitor once accurately placed, or by using a tape measure. Each participant will be asked to wear the activPAL™ professional physical activity logger for seven consecutive days at each time point to establish if there have been any changes in participants' activity levels during the course of the trial. Data will be explored to see if this device provides a useful outcome tool to measure free-living activity in CLBP, when compared with the more conventional daily activity logbook.

#### Daily activity/analgesic intake log-book

Participants will be asked to keep a logbook of their daily analgesic intake along with any changes in the strength/type of medication taken for their LBP. Daily exercise/activity will also be recorded in this logbook for comparison with the data generated by the activity monitor, along with any other treatment received. In addition, this logbook will be used by participants in treatment group A to record how often they manually stimulate the stud needles during each 48-hour auricular acupuncture treatment.

#### Holistic Complementary and Alternative Health Questionnaire

This is an 11-item scale, with six items relating to beliefs about the scientific validity of CAM, and five to beliefs about holistic health (HH) [36]. It is reported to have good test-retest reliability, and internal validity. Responses to each item are made using a six-point response format (strongly agree – strongly disagree). This will be collected at baseline and six months after randomisation to establish if there have been any changes in participants' beliefs of CAM and HH.

#### Fear-Avoidance Beliefs Questionnaire (FABQ)

This is a 16 item self-report questionnaire that specifically focuses on participants' beliefs about how physical activity (5 items) and work (11 items) affect their low back pain [37]. These data will be collected at baseline, at weeks 7 and 13, and 6 months after randomisation. The treating physiotherapists will be made aware of the results of the FABQ prior to commencing the exercise programme, in order to adapt the PEP accordingly. In order to limit respondent burden, participants will only be asked to complete the physical activity section (five items). This decision was made following analysis of pre-pilot data that showed low work section scores. A similar method was used in the UK BEAM trial [24].

#### Back Beliefs Questionnaire

This questionnaire [38] was developed with the aim of measuring an individual's beliefs about the inevitable aspects of the future as a consequence of low back pain. Higher scores indicate more positive beliefs, and less likelihood of absence from work. It consists of 14 questions, five of which are irrelevant and only included to distract the patient (Questions 4, 5, 7, 9, 11). The overall score is generated from the remaining nine questions. In order to limit respondent burden the participants will only be asked to complete the nine questions used to generate the overall score (therefore questions 4, 5, 7, 9, 11 will be omitted). A similar method was used in the UK BEAM trial [24].

#### General Perceived Self-efficacy Scale

The construct of Perceived Self-efficacy is the belief that one can perform novel or difficult tasks, or cope with adversity in various domains of human functioning [39]. Self-efficacy is an important factor in the self-management of chronic conditions and is highly correlated with disability [40]. This scale consists of ten items to assess this construct. In samples from 23 nations, Cronbach's alphas ranged from 0.76 to 0.90 [37].

#### EuroQol-5D

The EuroQol-5D [41] is a self-administered questionnaire that assesses the participant's health-related quality of life using a core set of five health-related quality of life items [42]. Its validity and reliability are supported, and it has been recommended for use in low back pain research [43]. For the UK population, an average weighted health index of 0.86 and self-rated health status of 82.48 have been reported in the literature [44]. Use of this outcome along with the information collected on use of health care resources will facilitate a cost-utility analysis of the trial interventions.

#### APEP Participants' Use of Health Care Resources

This questionnaire was designed by the trial team, in conjunction with a Health Economist.

#### Exit Questionnaire

Patient satisfaction is an important outcome within the field of healthcare. Since there is no single measure preferred within the literature [29], a general assessment of satisfaction [13] will be included in the exit questionnaire. The perceived benefit of treatment to participants, in relation to whether they achieved their initial treatment goals or not, along with any change in analgesic intake, will also be collected by this questionnaire. This will be collected 6 months after randomization only.

### Sample size

No sample size calculation has been carried out as this study aims to identify the referral rates of CLBP participants within the catchment area of the study site. Recruitment will be on a consecutive basis from participating practices, physiotherapy waiting lists and the general public, and will enable the researchers to estimate expected recruitment rates from each of these sources for the main RCT. The researchers aim to enrol eight cohorts of participants, each containing a maximum of n = 10 participants (total n = 80 participants), with randomisation to treatment taking place by cohort. Based on the results of previous trials investigating exercise or acupuncture for LBP, a 30% drop-out rate is anticipated between the beginning of treatment and the follow-up, so it is anticipated that n = 56 participants will complete the trial. The trial statistician is satisfied that this sample size will be sufficient to refine the study protocol, perform a power analysis, and establish the number of participants needed for the main RCT.

### Randomisation

Recruitment and flow of participants through the trial is represented in Figure 1. In accordance with recognised procedure, a computer-generated random allocation sequence will be prepared centrally prior to participant enrolment. This sequence will be used to allocate participants by cohort to one of two treatment groups-A or B. Treatment group A will receive PEP plus manual auricular acupuncture. Treatment group B will receive PEP alone.

The random allocation sequence will be generated by the trial statistician who will not be involved in the administration of treatment or collection of outcomes. The trial statistician will also ensure concealment of treatment allocation by placing individual random assignments into serially numbered sealed opaque envelopes.

### Blinding

This is a single-blind feasibility RCT; all outcomes will be collected by a blinded outcome assessor. It is not possible to blind either participants or physiotherapy practitioners to treatment due to the interventions under investigation.

### Qualitative assessment of this feasibility study

This will be conducted at the end of the treatment sessions (structured exercise ± manual auricular acupuncture) either at week 13 or week 25, depending on availability of the participants, in an attempt to improve the design, implementation and acceptability of the main RCT.

#### Participant Focus Groups

Participants will be invited to attend a focus group discussion. Each group will consist of a maximum of eight participants and will take place over a two-hour period. A 'clue and cue process', using a checklist of topics, will be used to ensure that the same basic areas are covered, but allowing any issues of importance to the participants to emerge. The main areas to be explored will be participants' interpretation of study information and documentation, their experiences, expectations and satisfaction with the programme of care, and acceptability of being involved in the trial. Sessions will be moderated by an experienced focus group moderator, audio-tape recorded, and field notes will be prepared by another member of the research team, not involved in the day-to-day running of the trial. Interviews will be transcribed, and interpretation, synthesis and data reduction undertaken independently by two members of the research team.

#### General Practice Interviews

An independent interviewer will also conduct one to one interviews with general practitioners and other staff, within each participating general practice, to explore strategies to improve referral and recruitment to the trial, and to assess their views of the overall value of the study to the National Health Service. Interviews will be audiotape recorded, transcribed and analysed as for the focus groups above.

### Statistical methods

Since this is a feasibility study, significance tests will not be performed or reported, and treatment effects will be represented by point estimates and confidence intervals. For each outcome variable ANCOVA will be performed on the data, conditioning on the baseline value. Trends over time and time-by-treatment interactions will be explored using repeated measures ANOVA. The influence of treatment preference on outcomes will also be explored. For all analyses, the residuals will be examined for evidence of non-normality. If substantial evidence of non-normality is found, appropriate alternative (non-parametric) tests will be applied, and specified as the primary analysis method in the main study. The cluster randomised nature of the design will be accounted for in the analysis by fitting a mixed effects model. Monthly recruitment rates and ratio of number screened: number enrolled will be tabulated. This information will be used to help select the recruitment period and number of centres for the main RCT. The assessment of participant satisfaction will be tabulated, as will any recorded difficulties experienced by the participants or therapists. This information may be used to modify the interventions used in the main RCT. If analgesic use differs substantially between the two groups, consideration will be given to using this as a primary outcome variable in the main RCT.

## Discussion

LBP can be a chronic problem in which activity along with pharmacological pain control is advocated to help foster active self-management strategies during recurrent episodes [1]. However, people with LBP often do not adhere to this advice either because of fear that activity will increase their pain and/or a lack of faith that medication could control activity related pain [7].

Acupuncture is commonly used by the general public for musculoskeletal pain in the UK and elsewhere [45-47]. Recent evidence suggests that stud auricular acupuncture may provide a means of allowing people with acute and chronic pain to manage their pain [14-18] but this has yet to be shown for LBP. This feasibility study is testing the trial procedures in preparation for a main trial; if we demonstrate clinically important adjuvant effects of auricular acupuncture to our exercise programme, this will provide the rationale for a fully powered trial. In addition to the quantitative aspects of this study, this pilot will also be informed by qualitative exploration of the conduct of this trial and the intervention packages. This information will be used to inform the conduct of the trial as it progresses, and also inform a main trial if the intervention effect sizes indicate that this is worthwhile.

## Competing interests

The author(s) declare that they have no competing interests.

## Authors' contributions

SMcD, DW, DL, DB, DH, AD, JP, and IB were involved in developing the original idea for funding and were co-applicants on the successful funding proposal. All authors participated in development of research protocols and design of the study. All authors read and approved the final manuscript.

## Pre-publication history

The pre-publication history for this paper can be accessed here:


